# Medical Management of Hereditary Optic Neuropathies

**DOI:** 10.3389/fneur.2014.00141

**Published:** 2014-07-31

**Authors:** Chiara La Morgia, Michele Carbonelli, Piero Barboni, Alfredo Arrigo Sadun, Valerio Carelli

**Affiliations:** ^1^UOC Clinica Neurologica, IRCCS Istituto delle Scienze Neurologiche di Bologna, Ospedale Bellaria, Bologna, Italy; ^2^Unità di Neurologia, Dipartimento di Scienze Biomediche e NeuroMotorie (DIBINEM), Università di Bologna, Bologna, Italy; ^3^Studio Oculistico d’Azeglio, Bologna, Italy; ^4^Istituto Scientifico San Raffaele, Milan, Italy; ^5^Doheny Eye Institute, University of California Los Angeles, Los Angeles, CA, USA

**Keywords:** optic nerve, LHON, OPA1, DOA, hereditary, optic atrophy, leber, mitochondria

## Abstract

Hereditary optic neuropathies are diseases affecting the optic nerve. The most common are mitochondrial hereditary optic neuropathies, i.e., the maternally inherited Leber’s hereditary optic neuropathy (LHON) and dominant optic atrophy (DOA). They both share a mitochondrial pathogenesis that leads to the selective loss of retinal ganglion cells and axons, in particular of the papillo-macular bundle. Typically, LHON is characterized by an acute/subacute loss of central vision associated with impairment of color vision and swelling of retinal nerve fibers followed by optic atrophy. DOA, instead, is characterized by a childhood-onset and slowly progressive loss of central vision, worsening over the years, leading to optic atrophy. The diagnostic workup includes neuro-ophthalmologic evaluation and genetic testing of the three most common mitochondrial DNA mutations affecting complex I (11778/ND4, 3460/ND1, and 14484/ND6) for LHON and sequencing of the nuclear gene *OPA1* for DOA. Therapeutic strategies are still limited including agents that bypass the complex I defect and exert an antioxidant effect (idebenone). Further strategies are aimed at stimulating compensatory mitochondrial biogenesis. Gene therapy is also a promising avenue that still needs to be validated.

## Introduction

Hereditary optic neuropathies are a heterogeneous group of diseases, which may be either an isolated optic neuropathy or optic neuropathy in the context of a more complex multi-systemic disease. The estimated minimum prevalence of hereditary optic neuropathies is about 1 in 10,000 (1).

Optic neuropathy, as part of a more complex spectrum of clinical symptoms, is common in hereditary neurodegenerative disorders with evidence of mitochondrial dysfunction [for a review see Ref. (2)] such as OPA1 “plus” syndrome (3), mitochondrial encephalomyopathy lactic acidosis and stroke-like syndrome (MELAS) (4), LHON/dystonia/MELAS/Leigh overlapping syndrome (5, 6), myoclonic epilepsy with ragged red fibers (MERRF) (7), POLG1-related syndrome (8), Friedreich ataxia (9), Charcot–Marie Tooth type 2A (10), Mohr–Tranebjerg syndrome (11), SPG7 syndrome (12), Wolfram syndrome (13), spinocerebellar ataxias (14), and DNMT1-related disorders (15).

The main non-syndromic hereditary optic neuropathies are Leber’s hereditary optic neuropathy (LHON) and dominant optic atrophy (DOA) and both are primary mitochondrial hereditary optic neuropathies (16). Other rare recessive or X-linked forms of optic neuropathy are also described, in most cases not yet resolved from the genetic point of view.

## Genetic Basis of Isolated Optic Neuropathies

Leber’s hereditary optic neuropathy is a maternally inherited disorder and in 90–95% of cases it is due to one of three mitochondrial DNA (mtDNA) mutations (11778/ND4, 3460/ND1, and 14484/ND6) (17). Other rare mtDNA mutations have been reported in association with LHON (18). These rare mutations may need testing for, by complete sequence of mtDNA, if the clinical suspicion of LHON is high and a maternal inheritance is obvious.

Dominant optic atrophy, in about 70% of cases, is due to mutations in the *OPA1* gene. Three other loci have been associated with autosomal DOA and designated as *OPA4* (19), *OPA5* (20), and *OPA8* (21). Furthermore, heterozygous mutations in the *OPA3* gene have been associated with DOA and cataract (22), as well as heterozygous mutations in the *WSF1* gene have been reported in association with DOA and hearing loss (23). Both *OPA3* and *WSF1* genes are mostly associated with recessive mutations leading to Costeff (24) and Wolfram syndromes, respectively. Additional loci have been identified in X-linked (*OPA2*) (25) and recessive (*OPA6* and *OPA7*) hereditary optic neuropathies, the latter recently associated with mutations in *TMEM126A* (26, 27).

The next sections will focus on LHON and DOA, the most frequent and prototypical hereditary optic neuropathies, for which an extensive body of studies is available.

## Clinical Presentation of LHON/DOA and Clinical Workup

**Figure 1:**
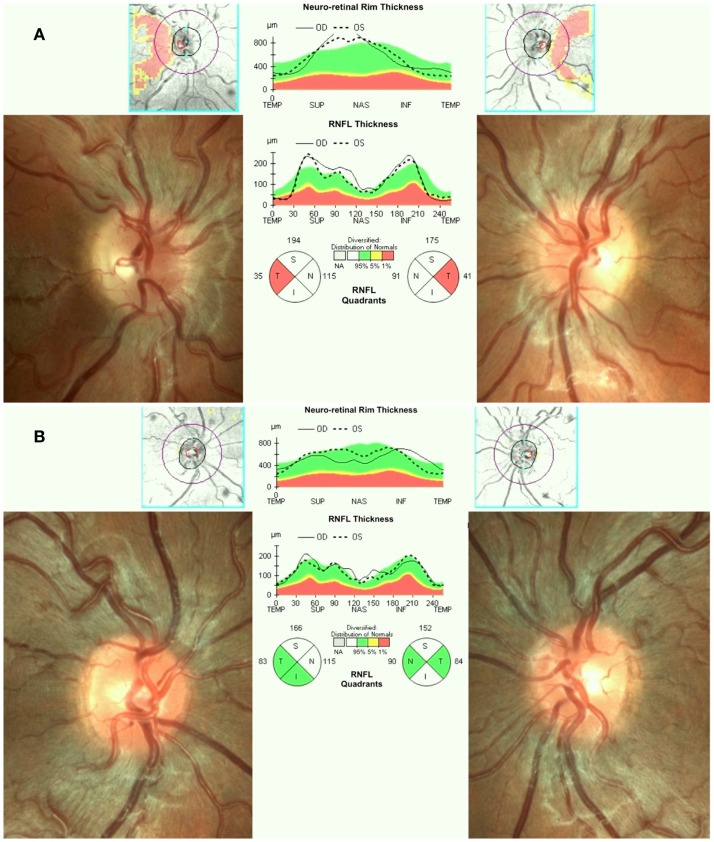
**(A)** Fundus and OCT in the acute-phase of LHON. Fundus exam shows moderate swelling of the retinal nerve fibers mainly involving the superior and inferior arcades around the optic disk associated with loss of fibers in the papillo-macular bundle (temporal pallor), retinal vessels tortuosity, and telangiectasias. Optical coherence tomography (OCT) shows retinal nerve fiber layer thinning of the temporal quadrant with thickening of all the other quadrants. **(B)** Fundus and OCT of a LHON carrier. Fundus examination reveals mild swelling of the superior and inferior retinal nerve fibers and microangiopathy. OCT demonstrates retinal nerve fiber layer thickening more evident in the supero-nasal (OD) and supero-inferior (OS) quadrants.

Leber’s hereditary optic neuropathy typically affects young adult males presenting with unilateral or bilateral subacute/acute painless loss of central vision. The clinical course typically involves a rapid worsening of visual acuity over a few weeks (nadir reached in about one to 6 months) and subsequent stabilization of visual loss, entering the chronic phase. Loss of vision can be very severe leading to legal blindness in most cases (17). In the acute phase, there is bilateral sequential vision loss, significant impairment of color vision, and usually maintenance of the pupillary light reflex. Fundus examination shows, in the acute phase, pseudoedema of the optic disk and retinal nerve fibers initially involving mainly the inferior and superior arcades with teleangectias (Figure 1A) and temporal or diffuse optic disk pallor in later stage of the disease. Visual fields reveal central or cecocentral scotoma, which progressively worsens over time (28). Optical coherence tomography (OCT) demonstrates an initial thickening of the temporal and inferior peripapillary fibers, which is followed by the thickening of the superior and, lastly, of the nasal fibers (Figure 1A). The temporal fibers are lost first and the nasal fibers are the most spared (29). Fluorescein angiography in the acute phase usually fails to show leakage of fluorescein (hence pseudo disk edema) and this is a useful marker for differentiating LHON from inflammatory optic neuropathies with true disk edema that causes fluorescein leakage (28). Another useful clinical sign, which distinguishes LHON from optic neuritis (the most important clinical differential diagnosis for LHON) is the usual absence of a relative afferent pupillary defect. The maintenance of the pupillary light response is partly due to the bilateral symmetry of the disease process and partly to the relative sparing of melanopsin retinal ganglion cells (mRGCs) in LHON, as revealed by post-mortem investigations (30). These cells are about 1% of the total RGCs and they contribute mainly to circadian photo-entrainment, but through the retinal projections to the olivary pretectal nucleus (OPN), they also regulate the pupillary light reflex. Clinical studies demonstrated the maintenance of the pupil responses in LHON patients (31, 32). The diagnostic workup in LHON also may include other neurophysiologic tests such as visual evoked potentials (VEPs) that reveal delayed latency and reduced amplitude of cortical responses and pattern electroretinograms (PERGs), which demonstrate reduced amplitude and delayed latency of responses (33).

Some LHON patients may spontaneously recover vision during the clinical course (34). The rate of spontaneous recovery is variable for the three LHON mutations being 4–20% for 11778/ND4 and 3460/ND1 mutation, and 50% or more for the 14484/ND6 (34, 35). This recovery may occur after the nadir of the disease at about 6–8 months and up to 5 years after the onset (1).

The family history is very helpful since it often reveals the presence of other affected individuals along the maternal lineage, in prevalence males.

Leber’s hereditary optic neuropathy is due in the large majority of cases (90–95%) to the three classical LHON mutations, which are homoplasmic (i.e., all the mtDNA copies harbor the mutation) in most of the families. Despite the genetic risk of disease seems to be equal among all individuals along the maternal lineage, the penetrance is incomplete and the disease affects prevalently males (17). Genetic counseling implies in these patients that the offspring of a woman carrying the LHON mutation will harbor the mutation, whereas offspring of males are spared. The probability to become affected is roughly 10% in women and 50% in men carrying the 11778/ND4 LHON mutation (17). The reason for the lower penetrance in females is possibly related to the protective role exerted by estrogens on cellular metabolism (36). The risk of recurrence for individuals younger than 50 years of age and maternally related to the index case has been calculated by Harding and colleagues (37). A specific mtDNA background, haplogroup J, due to recurrence of non-synonymous variants affecting complex I and III subunit genes, has been associated with higher penetrance of the mutations 11778/ND4 and 14484/ND6 (38).

Other known environmental risk factors for LHON are smoking, alcohol, and the use of some antibiotics (i.e., macrolids, aminoglicosides, ethambutol, isoniazide, and linezolid) (39, 40). Clinical counseling should include avoiding these risk factors.

Though usually LHON presents as an isolated optic neuropathy, in a few cases, there may be the coexistence of additional signs and symptoms, such as myoclonus, MS-like features, epilepsy, heart conduction defects, peripheral neuropathy, dystonia, and myopathy (41).

Other ancillary tests that have been shown useful to complete the clinical characterization of LHON patients include brain and muscle magnetic resonance spectroscopy (42, 43) and lactic acid after standardized exercise (44).

Asymptomatic carriers of LHON mutation may also have swollen retinal nerve fibers that can be seen on fundus examination and by OCT (45, 46) (Figure 1B), abnormalities in color vision (47), and in neural conduction along the visual pathways (33, 48).

Dominant optic atrophy usually affects both males and females in young childhood, and in the large majority of cases, is due to mutations in the *OPA1* gene (49, 50). There is incomplete penetrance and the classical clinical picture of DOA differs from LHON in that the former presents as a slowly progressive loss of vision with a smaller central or cecocentral scotoma (51). The clinical severity is very variable even within the same pedigree (52). As in LHON, OCT demonstrates preferential involvement of the papillo-macular bundle with a corresponding loss of fibers in the temporal sector of the optic disk and relative sparing of the nasal quadrant (53), which leads to temporal or diffuse optic atrophy. The main differential diagnosis for DOA is normal tension glaucoma, due to the frequent cupping of chronic DOA cases (54). Interestingly, OCT also demonstrates that optic disk size is reduced in DOA compared to controls and this might be explained by the role played by OPA1 in regulating developmental apoptosis and shaping optic nerve head morphology (55).

As for LHON, the clinical examination in DOA demonstrates the maintenance of the pupillary light reflex. This finding is corroborated by histological studies showing a relative sparing of mRGCs in DOA as well (30, 56).

VEPs may be normal or disclose a delayed latency and reduced amplitude of cortical responses (57).

Genetic counseling for DOA implies the rules of Mendelian autosomal dominant diseases and so the probability that offspring of an affected individual will inherit the mutation is 50% and this is equal for males and females. Prenatal genetic diagnosis is feasible, as well as pre-implantation diagnosis, thus providing the option to avoid transmission of the mutant *OPA1* allele to offspring of an affected parent. As for LHON, DOA also may present a “plus” phenotype, which includes deafness, chronic external ophthalmoplegia, peripheral neuropathy, cerebellar atrophy, and mitochondrial myopathy (3, 58). In these cases, muscle biopsy histology and histoenzymology may disclose the hallmarks of mitochondrial myopathy with cytochrome *c* oxidase negative fibers, whereas molecular analysis will reveal the pathological accumulation of multiple mtDNA deletions (58). Moreover, these patients often present increased lactic acid levels after standardized exercise. The OPA1 mutations associated with the “plus” phenotype are in most cases missense mutations affecting the GTP-ase domain of the OPA1 protein (58), as opposed to the majority of mutations associated with non-syndromic DOA, mostly predicted to induce haploinsufficiency.

## Follow-Up Management

Patients with hereditary optic neuropathies should be followed regularly during the disease course, particularly in the acute/subacute phase. The most important exams for the clinical follow-up of these patients are color vision, visual fields, visual acuity, and OCT.

**Figure 2:**
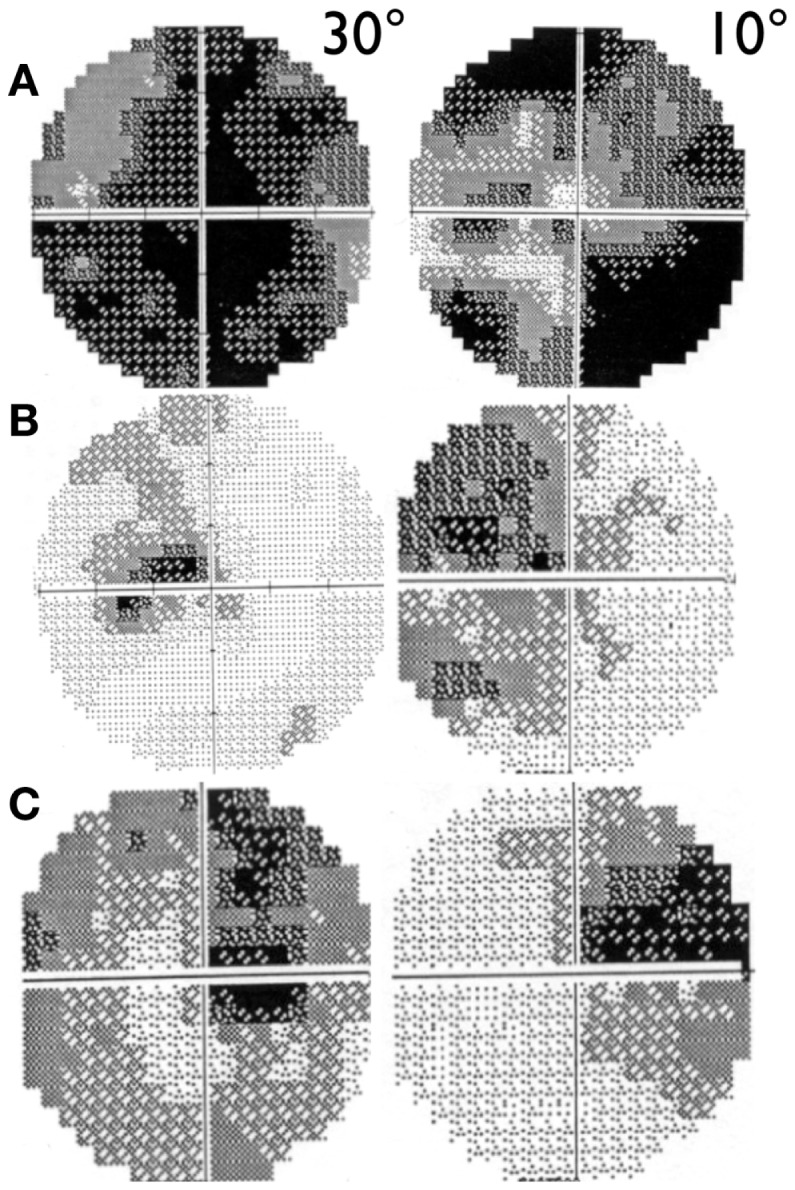
**30° and 10° visual field pattern of recovery in LHON**. **(A)** Example of scotoma fenestration (OS) in a LHON patient carrying the 14484/ND6 mutation **(B)** Example of scotoma contraction (OS) in a LHON patient carrying the 11778/ND4 mutation and **(C)** Example of both scotoma fenestration and contraction (OD) in a LHON patient carrying the 11778/ND4 mutation.

Visual acuity is usually poor in LHON and some patients may end with bare light perception. DOA patients present a more variable clinical severity (52). Visual fields usually deteriorate from small to very large central scotoma in LHON and may remain small and stable in DOA over long periods of time (59). In LHON, visual fields may sometimes show reduction in size of the scotoma or its fenestration (60), both leading to an improvement in visual acuity (Figure 2). However, spontaneous visual recovery has never been described in DOA (61). Only one rare OPA1 mutation has been associated with a reversible LHON-like visual loss (62). A component of the visual improvement in LHON can be the adaptive phenomenon of eccentric fixation (63).

## Clinical Significance

Although diseases as LHON and DOA are rare, they constitute a terrible tragedy for the patients who go blind and for their families. Unlike most common causes of blindness, these hereditary optic neuropathies are not associated with old age. Generally, patients with LHON and DOA loose their vision before young adulthood and the disease tend to be bilaterally symmetrical. Hence, typical patients will loose their vision as a teenager or in their 20s and face a lifetime of blindness and disability. Parents and siblings are often part of the process of diagnosis and management.

## Therapeutic Options

Therapeutic options for hereditary optic neuropathies are still limited (64) and up to now only one randomized double-blind placebo-controlled study with idebenone in LHON has been concluded (65).

Idebenone (a quinone analog of coenzyme Q10) has been reported to be effective in increasing the rate of recovery in LHON especially in patients with discordant visual acuity and if the patients are treated early in the disease course and for a long time (35, 65). These findings may suggest that there is a specific window of opportunity for increasing the rate of visual recovery with idebenone treatment. Coenzyme Q10 and idebenone are able to bypass the complex I defect caused by the LHON mutation and permit the transfer of electrons from NADH directly to complex III (66). A new double-blind placebo-controlled trial with idebenone in LHON is about to start with a longer follow-up and different design predefined primary end-points and dosages (Yu-Wai-Man, personal communication). Idebenone treatment has also been reported to ameliorate color vision in LHON (67).

Very preliminarily, encouraging results with idebenone treatment have been also reported in DOA patients (61) for whom spontaneous recovery has not been previously described. The rationale for the use of idebenone in DOA is that also in OPA1-related DOA, there is a complex I defect as in LHON (68).

More recently, other drugs are under investigations, such as the novel quinone molecule, alfa-tocotrienol EPI-743, which has shown promising results in an open-label trial on a limited group of LHON patients (69). These results need to be confirmed in larger controlled trial.

Other therapeutic strategies for LHON may include the use of drugs that might stimulate mitochondrial biogenesis, which has been demonstrated as a possible compensatory mechanism for LHON and other mitochondrial disorders (70). These include bezafibrate and rosiglitazone and novel molecules such as resveratrol and AICAR (5-aminoimidazole-4-carboxamide ribonucleoside) (71).

For a variety of reasons, gene therapy is a promising avenue for LHON treatment. The eye is easily accessible and provides a privileged space into which inject a viral vector. The most studied approach in LHON has been the AAV-mediated nuclear allotopic expression of mtDNA-encoded genes delivered to mitochondria (72, 73). This approach has been used in mouse models of LHON by the intravitreal delivery of an AAV vector carrying the wild-type ND4 (74, 75). This approach is now starting to be applied in LHON patients. However, it remains somehow controversial in terms of feasibility and efficiency (76). Other strategies include the direct delivery within mitochondria of the whole mtDNA or of an AAV vector containing the mtDNA-encoded ND4 subunit gene (77). These approaches are extremely promising but need to be validated.

For DOA, being a nuclear-encoded disease, gene therapy strategy should be more straightforward and feasible, as demonstrated by the recent encouraging results obtained with the gene therapy in Leber’s congenital amaurosis (78, 79). A proof of principle that genetically determined RGCs loss can be rescued by an AAV-based strategy with intravitreal delivery of the viral vector has been provided in an AIF mouse model, the so-called Harlequine mouse (80). There is issue of packaging, the 30 exons of the *OPA1* gene into the AAV. Another problem to solve is which isoform of the OPA1 gene should be used as the most efficient to rescue the phenotype in RGCs. Currently, the specific role played by the eight OPA1 isoforms has not yet been systematically investigated. Another critical point is that the delivery of wild-type *OPA1* to RGCs would likely complement haploinsufficiency mutations but more investigations are needed on the purported dominant negative mechanism of missense mutations. Nonetheless, gene therapy in DOA is a realistic objective, which will be pursued in the next future, providing some hope for treatment of DOA patients.

## Conflict of Interest Statement

Dr. Alfredo Arrigo Sadun is the PI on unrestricted research grants from Edison Pharmaceuticals. Dr. Valerio Carelli received reimbursements of travel expenses for consulting activities from Edison Pharmaceuticals. All other co-authors have no conflict of interest.
